# Higher circulating α-carotene was associated with better cognitive function: an evaluation among the MIND trial participants

**DOI:** 10.1017/jns.2021.56

**Published:** 2021-08-16

**Authors:** Xiaoran Liu, Klodian Dhana, Jeremy D. Furtado, Puja Agarwal, Neelum T. Aggarwal, Christy Tangney, Nancy Laranjo, Vincent Carey, Lisa L. Barnes, Frank M. Sacks

**Affiliations:** 1 Rush Institute for Healthy Aging, Rush University Medical Center, Chicago, IL, USA; 2 Department of Internal Medicine, Rush University Medical Center, Chicago, IL, USA; 3 Department of Nutrition, Harvard School of Public Health, Boston, MA, USA; 4 Rush Alzheimer's disease Center, Rush University Medical Center, Chicago, IL, USA; 5 Department of Neurology, Rush University Medical Center, Chicago, IL, USA; 6 Department of Preventive Medicine, Rush Medical College, Chicago, IL, USA; 7 Department of Clinical Nutrition, Rush College of Health Sciences, Chicago, IL, USA; 8 Channing Division of Network Medicine, Department of Medicine, Brigham and Women's Hospital, Boston, MA, USA; 9 Harvard Medical School, Boston, MA, USA; 10 Department of Nutrition, Harvard School of Public Health, Boston, MA, USA

**Keywords:** α-Carotene, Carotenoids, Cognitive function, Objective nutrient markers, Plasma, AD, Alzheimer's disease, FFQ, food frequency questionnaire, MIND, Mediterranean-DASH Intervention for Neurodegenerative Delay, MDLs, minimum detection limits

## Abstract

There is emerging evidence linking fruit and vegetable consumption and cognitive function. However, studies focusing on the nutrients underlying this relationship are lacking. We aim to examine the association between plasma nutrients and cognition in a population at risk for cognitive decline with a suboptimal diet. The Mediterranean-DASH Intervention for Neurodegenerative Delay (MIND) trial is a randomized controlled intervention that examines the effects of the MIND diet to prevent cognitive decline. The primary outcome is global cognition. A multivariate linear model was used to investigate the association between blood nutrients and global and/or domain-specific cognition. The model was adjusted for age, sex, education, study site, smoking status, cognitive activities and physical activities. High plasma α-carotene was associated with better global cognition. Participants in the highest tertile of plasma α-carotene had a higher global cognition *z* score of 0⋅17 when compared with individuals in the lowest tertile (*P* 0⋅002). Circulating α-carotene levels were also associated with higher semantic memory scores (*P* for trend 0⋅007). Lutein and zeaxanthin (combined) was positively associated with higher semantic memory scores (*P* for trend 0⋅009). Our study demonstrated that higher α-carotene levels in blood were associated with higher global cognition scores in a US population at risk for cognitive decline. The higher α-carotene levels in blood reflected greater intakes of fruits, other types of vegetables and lesser intakes of butter and margarine and meat. The higher circulating levels of lutein plus zeaxanthin reflected a dietary pattern with high intakes of fruits, green leafy, other vegetables and cheese, and low consumption of fried foods. Objective nutrient markers in the blood can better characterize dietary intake, which may facilitate the implementation of a tailored dietary intervention for the prevention of cognitive decline.

## Introduction

Diet has been identified as one of the important modifiable lifestyle factors in preventing Alzheimer's disease (AD). Despite a modest improvement in dietary quality over the last two decades, about 46 % of US adults have an overall poor dietary quality; particularly, the intakes of fruits and vegetables are lower than the recommended amount^(1)^. Carotenoids are potent antioxidants, naturally occurring pigments found in red, yellow, orange and dark green fruits and vegetables^(2)^. Emerging evidence from epidemiological studies links fruit and vegetable consumption^(3,4)^ and higher levels of carotenoids with a lower risk of cognitive decline among older adults from different regions^(5–9)^. In observational studies, dietary intakes of nutrients are commonly assessed by a food frequency questionnaire (FFQ). The majority of observational studies examining the relationship between nutrients and risk of cognitive decline have been conducted in older adults, and changes in cognitive function are common in this population^(10)^. Changes or impairment in memory may attenuate the accuracy in reporting and recall^(11)^. Conversely, objective measures, i.e., circulating nutrient levels, can accurately reflect an individual's nutritional status and dietary quality independent of their memory and cognition status. However, previous investigations on circulating levels of carotenoids and cognition remain inconclusive^(12–18)^.

Moreover, a large body of evidence from randomized intervention trials on blood concentrations of carotenoids and cognitive performance was focused on β-carotene, the most common form of supplementation^(19)^. An intervention approach that uses supplements to prevent disease risk largely neglects the interactive or synergic effects of nutrients within the food matrix and its relationship with disease risk. However, there is a dearth of evidence on the association between different forms of ‘dietary’ carotenoids and cognition.

Therefore, in the present study, we will leverage the Mediterranean-DASH Intervention for Neurodegenerative Delay (MIND) trial to examine (1) the association between dietary intakes of carotenoids and global/domain-specific cognition using objective measurements of plasma concentrations and (2) how participants’ dietary patterns corresponded to their plasma levels of carotenoids.

## Method

### Study design

The present study is an evaluation of baseline blood nutrients and cognition among the MIND participants. A detailed study design of MIND was published previously^(20)^. In brief, the MIND study (NCT02817074) is an ongoing randomized, controlled intervention that examines the effects of the MIND diet with mild caloric reduction (250 kcal) *v*. the usual diet with a mild caloric reduction on cognition in a population at risk for AD. We recruited community-dwelling adults in the Boston and Chicago city areas who were 65–84 years of age, overweight, at risk for AD and eating a suboptimal diet. We collected data on sociodemographic factors, lifestyle and medical information. Participants received standardized physical and neurological exams. Blood samples were collected at baseline and several time points during the 3-year study period. Plasma nutrients were measured in a randomly selected subset of participants (*n* 295). The institutional review boards of the Harvard T.H. Chan School of Public Health and Rush University Medical Center approved the study protocol, and all participants provided written informed consent.

### Dietary nutrients assessment using the FFQ

At baseline, participants completed a modified 142-item FFQ from the Harvard FFQ online. The modified FFQ was validated for use in an older Chicago population^(21)^. Serving sizes were based on either natural portions or age-specified mean portions according to the oldest persons in a national diet survey. Participants’ dietary quality was assessed using a 14-item MIND diet questionnaire. A suboptimal diet was defined as a MIND score ≤8 out of 14.

### Nutrients assessment in the blood

Fasting blood samples were obtained at the baseline visits. Concentrations of lutein and zeaxanthin (combined), β-cryptoxanthin, lycopene, α-carotene and β-carotene in the plasma samples were measured using the method described by Hess^(22)^, with some modifications. Samples were quantitated by high-performance liquid chromatography (HPLC) on a Restek Ultra C18 150 mm × 4⋅6 mm column with 3 μm particle size encased in a Hitachi L-2350 column oven to prevent temperature fluctuations and equipped with a trident guard cartridge system (Restek Corp., Bellefonte, PA, USA). A mixture of acetonitrile, tetrahydrofuran, methanol and a 1 % ammonium acetate solution (68 : 22 : 7 : 3) was used as the mobile phase, at a flow rate 1⋅1 ml/min, using a Hitachi Elite LaChrom HPLC system comprised of an L-2130 pump in isocratic mode, an L-2455 Diode Array Detector (monitoring at 300 and 445 nm), and a programmable AS-2200 auto-sampler with a chilled sample tray. The system manager software (D-7000, Version 3⋅0) was used for peak integration and data acquisition (Hitachi, San Jose, CA, USA). The minimum detection limits in plasma were (μm/l) 3⋅86 for lutein and zeaxanthin (combined), 3⋅88 for β-cryptoxanthin, 5⋅44 for lycopene, 4⋅24 for α-carotene and 4⋅80 for β-carotene. Because lutein and zeaxanthin co-elute on the chromatogram, the two are grouped and presented as lutein and zeaxanthin (combined). For external quality control, our laboratory participates in the standardisation programme for carotenoid analysis of the National Institute of Standards and Technology USA.

### Cognitive assessment

The main outcome of the MIND study is the change in global cognitive function at baseline and year 3. In the present study, the exposures are the plasma carotenoids and the outcomes are global and domain-specific cognition at baseline. The global measure of cognitive function included a neuropsychological test battery of twelve performance-based tests. The test battery includes multiple tests for each of four cognitive domains. For episodic memory, we used immediate and delayed recall of the East Boston story^(23)^ and the Consortium to Establish a Registry for Alzheimer's Disease (CERAD) Word List learning, recall and recognition^(24)^. For semantic memory, we used category fluency^(24)^ and the Multilingual Naming Test^(25)^. For executive function, we used (Trails B)^(26)^ and the NIH toolbox flanker test^(27)^. For perceptual speed, we used (Trails A)^(26)^, the Digit Symbol Substitution Test^(28)^, and the NIH toolbox pattern comparison test^(29)^. The global cognition score is created by converting raw scores on each test to *z* scores, and then averaging the *z* scores^(30)^, a method that has been used in many previous studies^(30–34)^.

## Statistics

Baseline characteristics are presented as mean and standard deviations (sds) for continuous variables, and as *n* (%) for categorical variables. We performed log transformation on data with skewed distribution. We examined blood nutrient markers as a categorical variable in tertiles where the lowest tertile for each nutrient was set as the reference with a *z* score of zero. Total carotenoids were calculated as the sum of α-carotene and β-carotene, the two main form of carotenoids. A multivariate linear model was used to examine the association between blood nutrient markers and global and domain-specific cognitive function. Estimated coefficients and their 95 % CIs were reported. We used Benjamini–Hochberg correction with a false discovery rate of 20 % to control the false discovery rate^(35)^. The multivariate model was adjusted for age, sex, smoking status (none, current and former), total cholesterol, study site, calorie intakes and cognitive activity. The cognitive activity score was derived from the average frequency of participation in various activities (e.g., reading, playing games, writing letters and visiting the library) and rated on a 5-point scale. The median of each tertile was used to assess the linearity of the association reported as *P* for trend. In the multivariate model, we further mutually adjusting α-carotene, β-carotene, β-cryptoxanthin, lutein plus zeaxanthin and lycopene to examine the independent association between each nutrient and cognitive function. After identifying the association between blood nutrients and cognition, we applied a partial correlation that accounted for age, BMI and total energy intakes to examine the associations between food intakes and blood nutrient status, which identified food items contributing to specific blood nutrients. We then used multivariate models to further adjust for income to determine how participants’ dietary patterns corresponded to plasma nutrient levels in tertiles. All data were analysed using the SAS system for Windows (Release 9.4; SAS Institute Inc., Cary, NC, USA).

## Results

The baseline characteristics of the study participants are presented in Table 1. Participants are predominantly Caucasian females with a mean age of 69⋅8 years. The mean plasma levels for total carotenoids were 451⋅5 (μg/l), 102⋅4 μg/l for α-carotene, 349⋅0 μg/l for β-carotene, 134⋅9 μg/l for β-cryptoxanthin, 205⋅9 μg/l for lutein and zeaxanthin (combined), and 448⋅9 μg/l for lycopene. The association between dietary-derived carotenoids and circulating levels is presented in Table 2. Dietary FFQ-derived carotenoids have a low-to-moderate correlation with plasma levels with a correlation coefficient range from 0⋅23 for lycopene (*P* < 0⋅0001) to 0⋅44 for β-cryptoxanthin (*P* < 0⋅0001).

**Table 1.** Baseline characteristics of participants*

Variables	*N* 295
Age (years)	69⋅8 ± 4⋅2
Sex (%)	
Female	67⋅8
Body mass index (kg/m^2^)	33⋅6 ± 5⋅6
Smoking status (%)	
Former	51⋅9
Never	45⋅8
Current	2⋅3
Education (%)	
Up to high school, high school equivalent	21⋅0
College degree	23⋅7
Post-graduate degree	55⋅3
Race (%)	
White	86⋅4
Black or African American	12⋅2
Other	1⋅40
Plasma carotenoids (μg/l)	
α-Carotene	102⋅4 ± 116⋅2
β-Carotene	349⋅0 ± 299⋅6
β-Cryptoxanthin	134⋅9 ± 105⋅8
Lutein plus zeaxanthin	205⋅9 ± 111⋅7
Lycopene	448⋅9 ± 193⋅7
Lipids (mg/dl)	
HDL-cholesterol	54⋅0 ± 16⋅9
Non-HDL-cholesterol	136⋅8 ± 39⋅7

* Values are means ± sd or percentages.

**Table 2.** Association between dietary-derived carotenoids and circulating levels of carotenoids

	Plasma levels					
FFQ-derived levels	Total carotenoids^a^	α-Carotene	β-Carotene	Lutein plus zeaxanthin	β-Cryptoxanthin	Lycopene
α-Carotene	0⋅25	0⋅33	0⋅20	0⋅10	0⋅07	0⋅11
	<0⋅0001	<0⋅0001	0⋅0004	0⋅09	0⋅24	0⋅05
β-Carotene	0⋅28	0⋅21	0⋅28	0⋅21	0⋅07	0⋅12
	<0⋅0001	0⋅0004	<0⋅0001	0⋅0004	0⋅23	0⋅04
Lutein plus zeaxanthin	0⋅17	0⋅10	0⋅19	0⋅24	0⋅10	0⋅10
	0⋅0029	0⋅07	0⋅001	<0⋅0001	0⋅09	0⋅08
β-Cryptoxanthin	0⋅17	0⋅15	0⋅16	0⋅05	0⋅44	0⋅14
	0⋅0034	0⋅01	0⋅005	0⋅35	<0⋅0001	0⋅02
Lycopene	0⋅03	0⋅08	0⋅01	0⋅01	−0⋅04	0⋅23
	0⋅61	0⋅16	0⋅90	0⋅81	0⋅45	<0⋅0001

a Sum of α-carotene and β-carotene.

### Plasma levels of carotenoids and global and domain-specific cognition

The association between plasma concentrations of total carotenoids and the higher global cognitive score was marginally significant (*P* for trend 0⋅09, Table 3). A closer investigation revealed that high plasma levels of α-carotene were associated with a higher global cognitive score (*P* for trend 0⋅001). In comparison with the individuals in the lowest tertile (T1) for plasma α-carotene, those in the middle tertile (T2) had a 0⋅13 higher global cognition score (*P* 0⋅04), and those in the highest tertile (T3) had a 0⋅17 higher global cognition score (*P* 0⋅002) (Table 3). The associations between β-carotene, β-cryptoxanthin, lutein and zeaxanthin, lycopene and global cognition were not statistically significant. To investigate the independent association between α-carotene and global cognition, we further adjusted levels of β-carotene, β-cryptoxanthin, lutein and zeaxanthin, and lycopene in the multivariate model. The positive association between α-carotene and global cognition remained unchanged (*P* 0⋅0074).

**Table 3.** Multivariate association between plasma concentrations of carotenoids in tertiles and global cognitive function

	Global cognitive function (*z* score) in tertile *β* (se)			
	T1	T2	T3	*P* for trend
Total carotenoids^a^				
Median (μg/l)	162⋅4	369⋅3	709⋅2	
Age-adjusted	Ref.	−0⋅06 (0⋅06)	0⋅14 (0⋅06)	0⋅01
Multivariate	Ref.	−0⋅10 (0⋅06)	0⋅06 (0⋅06)	0⋅09
α-Carotene				
Median (μg/l)	34⋅9	72⋅6	155⋅8	
Age-adjusted	Ref.	0⋅15 (0⋅07)*	0⋅20 (0⋅07)*	0⋅00
Multivariate	Ref.	0⋅13 (0⋅06)*	0⋅17 (0⋅07)*	0⋅01
β-Carotene				
Median (μg/l)	120⋅6	287⋅8	544⋅0	
Age-adjusted	Ref.	−0⋅08 (0⋅07)	0⋅14 (0⋅07)	0⋅05
Multivariate	Ref.	−0⋅11 (0⋅06)	0⋅10 (0⋅07)	0⋅08
β-Cryptoxanthin				
Median (μg/l)	53⋅6	102⋅4	215⋅9	
Age-adjusted	Ref.	0⋅02 (0⋅07)	0⋅06 (0⋅07)	0⋅33
Multivariate	Ref.	0⋅01 (0⋅06)	0⋅02 (0⋅07)	0⋅73
Lycopene				
Median (μg/l)	269⋅4	430⋅6	626⋅2	
Age-adjusted	Ref.	0⋅07 (0⋅07)	0⋅06 (0⋅07)	0⋅37
Multivariate	Ref.	0⋅07 (0⋅06)	0⋅06 (0⋅06)	0⋅33
Lutein and zeaxanthin				
Median (μg/l)	109⋅5	180⋅6	289⋅7	
Age-adjusted	Ref.	0⋅14 (0⋅07)	0⋅10 (0⋅07)	0⋅14
Multivariate	Ref.	0⋅11 (0⋅07)	0⋅05 (0⋅07)	0⋅45

Data are cognitive function *z* score, *β* (se).

A multivariate model was adjusted for age, sex, study site, smoking status (never, former and current), total cholesterol, calorie intakes and cognitive activity.

Benjamini–Hochberg correction with a false discovery rate of 20 % was used to control the false discovery rate.

a Sum of α-carotene and β-carotene.

* *P* < 0⋅05 when compared with the reference group.

There was a domain-specific association between α-carotene, lutein and zeaxanthin with semantic memory. Higher levels of α-carotene were associated with higher semantic memory scores (*P* for trend 0⋅007, Table 4). Individuals in the highest tertile (T3) of plasma α-carotene had a greater semantic memory score of 0⋅62 when compared with those in the lowest tertile (T1). Similar, circulating levels of lutein and zeaxanthin were positively associated with a semantic memory score (*P* for trend 0⋅01). Those with the highest plasma levels of (lutein and zeaxanthin) had 0⋅59 scores higher for semantic memory than those in the lowest tertile. Moreover, there was a tendency for a positive correlation between levels of α-carotene and episodic memory (*P* 0⋅06).

**Table 4.** Multivariate associations between plasma concentrations of carotenoids in tertiles and domain-specific cognition

	Domain-specific cognition			
	T1	T2	T3	*P* for trend
Executive functioning				
Total carotenoids	Ref.	−0⋅22 (0⋅31)	0⋅09 (0⋅31)	0⋅67
α-Carotene	Ref.	0⋅27 (0⋅30)	0⋅25 (0⋅31)	0⋅42
β-Carotene	Ref.	−0⋅35 (0⋅30)	0⋅11 (0⋅31)	0⋅83
Lutein and zeaxanthin	Ref.	0⋅36 (0⋅30)	−0⋅05 (0⋅30)	0⋅89
β-Cryptoxanthin	Ref.	−0⋅43 (0⋅30)	−0⋅02 (0⋅30)	1⋅00
Lycopene	Ref.	0⋅53 (0⋅30)	0⋅06 (0⋅30)	0⋅93
Perceptual speed memory				
Total carotenoids	Ref.	−0⋅25(0⋅22)	0⋅08 (0⋅23)	0⋅60
α-Carotene	Ref.	0⋅37 (0⋅22)	0⋅28 (0⋅23)	0⋅24
β-Carotene	Ref.	−0⋅22 (0⋅22)	0⋅14 (0⋅23)	0⋅63
Lutein and zeaxanthin	Ref.	0⋅31 (0⋅22)	−0⋅03 (0⋅23)	0⋅92
β-Cryptoxanthin	Ref.	−0⋅19 (0⋅22)	−0⋅06 (0⋅22)	0⋅81
Lycopene	Ref.	0⋅24 (0⋅22)	0⋅42 (0⋅22)	0⋅06
Episodic memory				
Total carotenoids	Ref.	−0⋅41 (0⋅43)	0⋅76 (0⋅45)	0⋅05
α-Carotene	Ref.	0⋅23 (0⋅44)	0⋅66 (0⋅45)	0⋅06
β-Carotene	Ref.	−0⋅43 (0⋅43)	0⋅74 (0⋅44)	0⋅43
Lutein and zeaxanthin	Ref.	0⋅34 (0⋅43)	0⋅40 (0⋅44)	0⋅56
β-Cryptoxanthin	Ref.	0⋅71 (0⋅43)	0⋅43 (0⋅43)	0⋅41
Lycopene	Ref.	0⋅11 (0⋅44)	0⋅08 (0⋅44)	0⋅91
Semantic memory				
Total carotenoids	Ref.	−0⋅12 (0⋅22)	0⋅19 (0⋅23)	0⋅33
α-Carotene	Ref.	0⋅23 (0⋅22)	0⋅62 (0⋅22)*	0⋅007
β-Carotene	Ref.	−0⋅12 (0⋅22)	0⋅30 (0⋅22)	0⋅28
Lutein and zeaxanthin	Ref.	0⋅57 (0⋅22)*	0⋅59 (0⋅22)*	0⋅01
β-Cryptoxanthin	Ref.	0⋅15 (0⋅22)	0⋅26 (0⋅22)	0⋅23
Lycopene	Ref.	−0⋅11 (0⋅22)	0⋅19 (0⋅22)	0⋅88

Data are cognitive function *z* score, *β* (se).

A multivariate model was adjusted for age, sex, study site, smoking status (never, former and current), total cholesterol, calorie intakes and cognitive activity. Benjamini–Hochberg correction with a false discovery rate of 20 % was used to control the false discovery rate.

*P < 0⋅05.

### Food consumption, dietary pattern and plasma levels of α-carotene

We first identified the association between plasma levels of α-carotene and global cognitive performance. We then investigated food groups and dietary patterns that contributed to the plasma α-carotene levels (Table 5). The consumption of fruits, nuts and vegetables other than green leafy vegetables (other types of vegetables) was associated with higher α-carotene levels in the blood (*P* < 0⋅05 for all). Specifically, the association between the intake of other types of vegetables and α-carotene was independent of other food groups (*P* 0⋅0032). The components of other types of vegetables are presented in Supplementary Table S1.

**Table 5.** Association between food intakes and plasma α-carotene levels

	Plasma α-carotene		Lutein plus zeaxanthin	
Food groups (serving/week)	Correlation coefficient	*P*-value	Correlation coefficient	*P*-value
Other vegetables	0⋅33	<0⋅0001	0⋅19	0⋅001
Green leafy vegetables	0⋅06	0⋅17	0⋅19	0⋅001
Fruits	0⋅26	0⋅002	0⋅10	0⋅07
Juice	0⋅04	0⋅78	−0⋅02	0⋅76
Berries	0⋅02	0⋅97	0⋅08	0⋅17
Nuts	0⋅12	0⋅09	0⋅005	0⋅93
Olive oil	0⋅04	0⋅66	−0⋅02	0⋅74
Whole grains	0⋅11	0⋅13	−0⋅03	0⋅57
Fish	0⋅06	0⋅79	0⋅03	0⋅65
Poultry	0⋅12	0⋅09	0⋅10	0⋅86
Meat	−0⋅14	0⋅02	−0⋅05	0⋅38
Butter and stick margarine	−0⋅13	0⋅03	−0⋅07	0⋅22
Cheese	0⋅09	0⋅26	0⋅12	0⋅03
Pastries and sweets	−0⋅12	0⋅03	−0⋅10	0⋅07
Fried foods	−0⋅06	0⋅79	−0⋅09	0⋅11

Multivariate model adjusted for age and BMI.

Regarding plasma levels of lutein and zeaxanthin, the consumption of green leafy vegetables (*P* 0⋅001), other types of vegetables (*P* 0⋅001) and cheese (*P* 0⋅03) was associated with higher lutein and zeaxanthin levels.

**Fig. 1.**
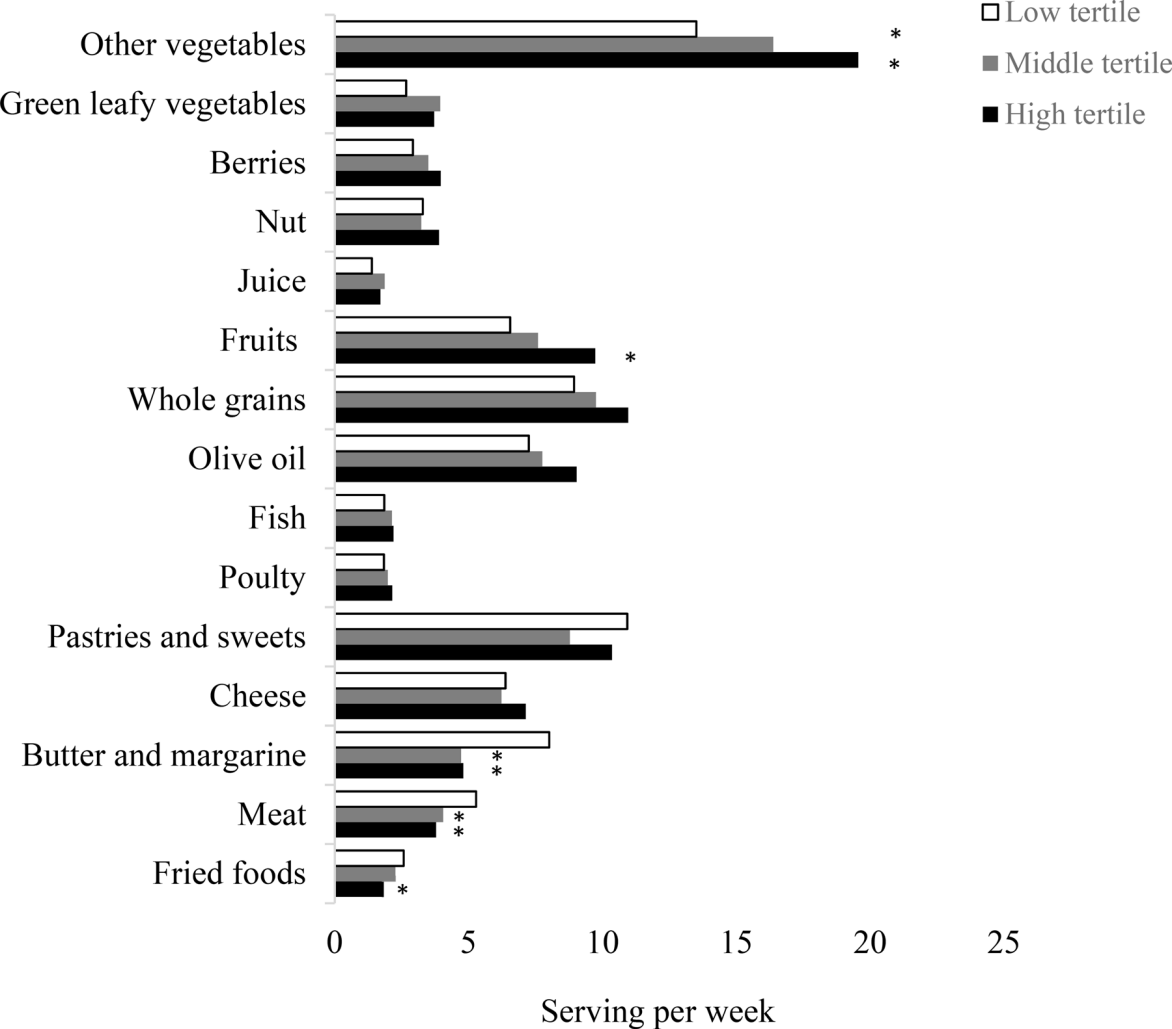
Participants’ baseline dietary pattern according to tertiles of plasma α-carotene levels. Data are average food consumption (serving per week). *P < 0⋅05 when compared to the reference group.

**Fig. 2.**
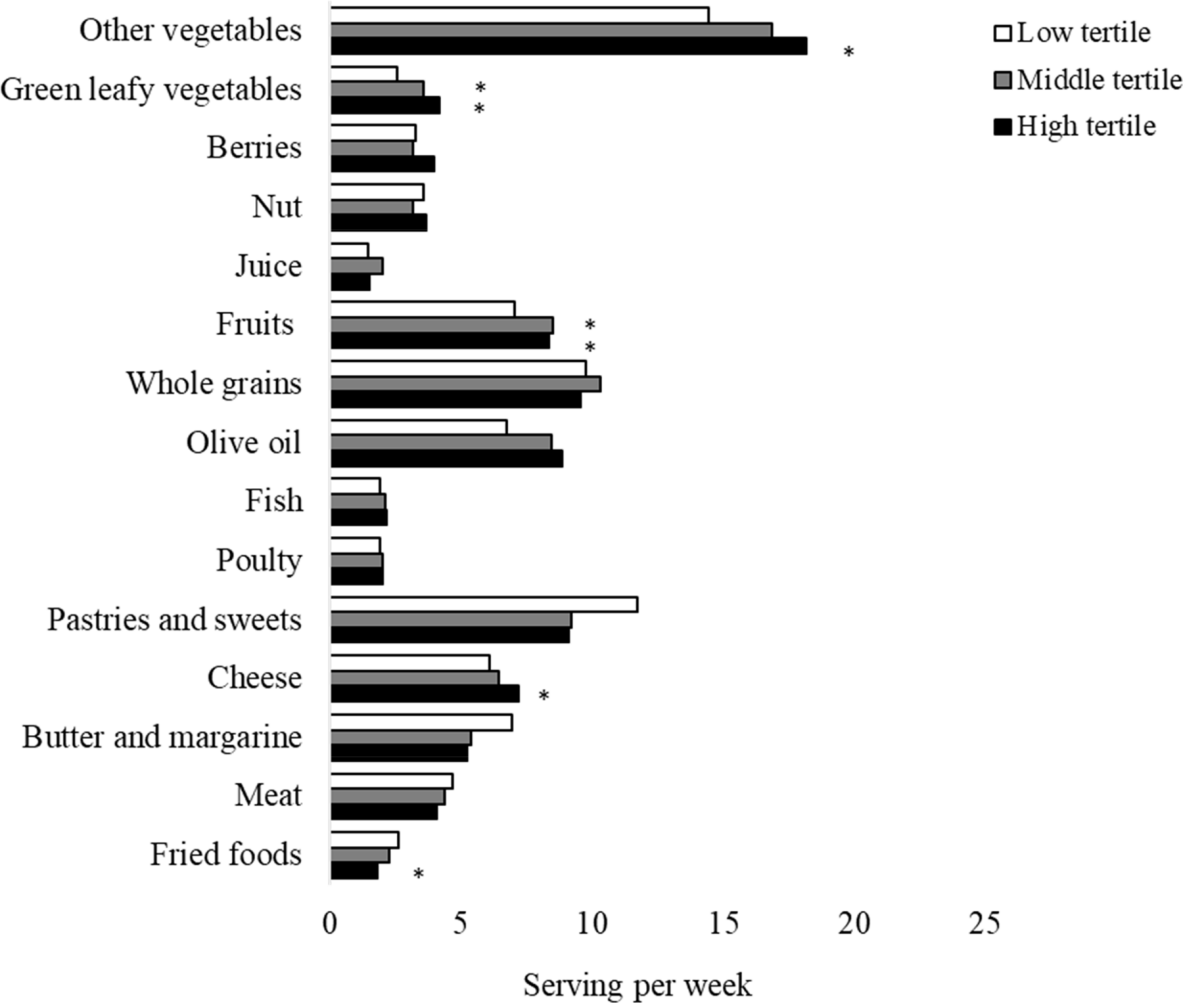
Participants' baseline dietary pattern according to tertiles of plasma lutein plus zeaxanthin levels. Data are average food consumption (serving per week). *p < 0.05 when compare to the reference group.

We then identified the dietary patterns corresponding to levels of plasma α-carotene (Fig. 1) and lutein plus zeaxanthin (Fig. 2). When comparing extreme tertiles, participants with the highest plasma α-carotene levels had significantly greater intakes of fruits (9⋅7 *v*. 6⋅6 serving/week, *P* < 0⋅05) and other types of vegetables (19⋅6 *v*. 13⋅5 serving/week, *P* < 0⋅0001), as well as lower consumption of meat (3⋅8 *v*. 5⋅3 serving/week, *P* < 0⋅0001), butter and margarine (4⋅8 *v*. 8⋅0 serving/week, *P* 0⋅0004), and fried foods (1⋅8 *v*. 2⋅6 serving/week, *P* 0⋅0028).

In comparison with participants with the lowest tertile of plasma lutein plus zeaxanthin, those with the highest plasma lutein and zeaxanthin levels had significantly higher consumption of other types of vegetables (18⋅2 *v*. 14⋅4 serving/week, *P* 0⋅0038), green leafy vegetables (4⋅2 *v*. 2⋅6 serving/week, *P* < 0⋅0001) and cheese (7⋅2 *v*. 6⋅1 serving/week, *P* 0⋅049), and lower consumption of fried foods (1⋅8 *v*. 2⋅6 serving/week, *P* 0⋅0051).

## Discussion

In the present study, we demonstrated that higher circulating levels of α-carotene were associated with higher scores in global cognition and semantic memory. Additionally, plasma lutein and zeaxanthin (combined) were associated with a higher score for semantic memory. The α-carotene levels in blood reflected of greater intakes of other types of vegetables in a US adult population at risk for AD dementia. The novelty of the study includes (1) the use of objective blood biomarkers assessing nutrient status in older adults and (2) the assessment is on circulating levels of nutrients from ‘dietary’ sources of carotenoids as oppose to supplementations.

Circulating carotenoids in the human body are obtained mainly through diet or antioxidant supplements^(2)^. Compared with self-reported data, objective nutrient biomarkers are not subjective to the potential errors due to cognitive decline. Changes in cognitive function are common in older adults^(10)^. However, there is limited research on the impact of cognitive function on the integrity of self-reported dietary data. One study conducted by Bowman et al. compared nutrients assessed by the FFQ and plasma nutrient biomarkers among older adults with or without mild cognitive impairment^(11)^. Their study demonstrated that (1) cognitive impairment attenuated the validity of FFQ and (2) the validity between estimates of FFQ and plasma nutrients varies according to the nutrient of interest^(11)^. Notebly, in individuals with mild cognitive impairment (MCI), estimated levels of carotenoids from FFQ did not correlate with plasma levels. Although their study was limited by a small sample size, their results are indicative that the influence of cognitive function on dietary assessment should be considered among older participants. Our study assessed the nutrients status using objective blood concentrations that were not susceptible to participants’ cognitive status. We examined the association between α-carotene and global cognition and identified the dietary components contributing to the circulating α-carotene levels that related to better cognitive performance. The independent relation between α-carotene and global cognition, lutein and zeaxanthin with semantic memory provided mechanistic insights regarding the potential roles of these nutrients in the prevention of cognitive decline.

The mechanisms at work include the antioxidant capacity of carotenoids potentially preventing neurodegeneration by reducing in oxidative stress^(36–38)^. Although direct mechanistic links between α-carotene and cognition are limited, a high plasma α-carotene concentration has been associated with a lower mortality risk in a nationally representative sample of US adults^(39)^. It is worth noting that because α-carotene is not widely available in supplement form^(40)^; therefore, it was hypothesised that these participants were obtaining their α-carotene from dietary sources, i.e., fruits and vegetables. Intakes of fruits and vegetables were linked to a decreased likelihood of cognitive decline^(3,4,41,42)^. Plasma α-carotene is highly correlated with the total consumption of fruits and vegetables, particularly carrots and other root vegetables^(43,44)^. Among participants from the Framingham Heart Study, 75 % of dietary α-carotene was from carrots^(45)^. Indeed, in the present study, the high α-carotene in the blood was reflective of a diet with greater intakes of a variety of root vegetables and peppers (i.e., carrots, sweet potatoes, yams, red, green, yellow peppers and squash) and fruits. Participants in the middle and highest tertiles for α-carotene had significantly higher consumption of vegetables other than green leafy vegetables and fruits, as well as lower consumption of meat, butter and margarine, and fried foods.

We observed a domain-specific association between lutein and zeaxanthin (combined) and semantic memory. In line with previous observations, plasma lutein and zeaxanthin (combined) was associated with better global cognition, memory and executive function in 4076 community-dwelling adults aged 50 years or older from Ireland^(46)^. Lutein and zeaxanthin account for 66–77 % of the total carotenoids accumulated in the brain tissue^(47–49)^. The current evidence supports the role of lutein in neural health during aging^(49)^. Although the molecular pathways that link lutein and zeaxanthin with cognitive function remain uncertain, several underlying mechanisms that suggest neuroprotective effects have been proposed. The brain is vulnerable to free radicals given its high metabolic activity and high concentration of polyunsaturated fatty acids^(50)^. Therefore, the antioxidant capacity of lutein could inhibit free radical formation preventing neuron damage from oxidative stress^(51)^. Lutein and zeaxanthin also activate the anti-inflammatory pathways reducing neuroinflammation, which is one factor contributing to AD's pathogenesis^(51,52)^. One intervention study evaluated the effects of lutein and DHA supplementation on cognitive function among women between 60 and 80 years without cognitive impairment. After 4 months, lutein (12 mg/d) improved long-term memory retrieval; the combination of DHA (800 mg/d) and lutein improved memory scores and rates of learning^(53)^. These exploratory findings add to the evidence base on the effects of antioxidants in improving cognition among older adults.

In the present study, we identified different dietary patterns corresponding to circulating levels of α-carotene, lutein and zeaxanthin. Compared with those with higher plasma levels of α-carotene had higher intakes of fruits, other vegetables, and lower butter and margarine, and meat consumption. Elevated plasma levels of lutein and zeaxanthin were associated with higher intakes of green leafy vegetables, other vegetables, cheese and lower fried foods. It is indicative that the domain-specific association between lutein and zeaxanthin and semantic memory might be attributable to a slightly different dietary pattern.

Our study has limitations. The study participants were overweight with a suboptimal diet, limiting the generalisability of the study results. However, obesity is prevalent in the USA, and nearly half US adults have a similarly poor dietary quality^(1)^. The cross-sectional nature of this investigation precluded any conclusion regarding causality. We cannot eliminate the possibility of reverse causality (i.e., the potential change in dietary habits due to changes in cognitive function in this population). Additionally, the cost of measuring objective nutrient biomarkers may limit their availabilities in population-based studies. The strengths of our study include that we used objective nutrient markers to assess participants’ nutrient status. The objective measurements are not susceptible to recall bias and can provide direct mechanistic links with global and domain-specific cognition among older adults. In the present study, we measured different forms of carotenoids from dietary sources, demonstrating the unique association between α-carotene and cognitive performance. The homogeneity of the study population may minimise certain residual confounding. Additionally, we used a neuropsychological battery of twelve performance-based tests to assess both global and domain-specific cognition.

## Conclusion

In a US population at risk for AD with a suboptimal diet, high levels of plasma α-carotene were associated with higher scores for global cognition, and episodic and semantic memory. Lutein and zeaxanthin (combined) were positively associated with better scores for semantic memory. A dietary pattern that featured greater consumption of vegetables other than green leafy vegetables and fruits corresponded to high α-carotene in blood was associated with higher cognition scores. Using blood nutrient levels as objective markers could characterize individuals’ dietary patterns, which could facilitate a targeted dietary intervention to prevent cognitive decline.

## Supplementary material

For supplementary material accompanying this paper visit http://dx.doi.org/10.1017/jns.2021.56. click here to view supplementary material
