# Supplementary material for: Higher circulating α-carotene was associated with better cognitive function: an evaluation among the MIND trial participants
Source: J Nutr Sci. 2021 Aug 16;10:e64. doi: 10.1017/jns.2021.56 (PMC8411267; doi:10.1017/jns.2021.56)
Supplement: Supplementary file 1 [file S2048679021000562sup001.docx]

**Supplementary Material**

**Table 1**. Component of other types of vegetables (weekly consumption)

|  | Serving/week |
| --- | --- |
| Tomatoes | 2.2±1.6 |
| String beans and green beans | 0.9±1.0 |
| broccoli | 1.2±1.2 |
| Beets | 0.4±0.6 |
| Corn | 0.6±0.7 |
| Peas or lima beans | 0.5±0.7 |
| Mixed vegetables | 0.6±1.0 |
| Green, red, yellow peppers | 1.2±1.3 |
| Yams, or sweet potatoes | 0.6±0.8 |
| Potatoes (bake, boil, mash) | 1.0±1.0 |
| Zucchini, squash, eggplant | 0.7±0.9 |
| Brussels sprouts | 0.4±0.6 |
| Mushrooms | 0.8±1.1 |
| Carrots cooked | 0.7±0.8 |
| Carrots raw | 1.0±1.3 |
| Celery | 0.8±1.1 |
| Onions | 2.1±2.0 |
| Cabbage or coleslaw | 0.6±1.0 |

^*^Values are means ± SDs

|  | (servings/week) |
| --- | --- |
| Other vegetables | 16.5±9.0 |
| Green leafy vegetables | 3.4±2.8 |
| Berries | 3.5±3.4 |
| Fruits | 7.96±5.1 |
| Nut | 3.5±2.9 |
| Olive oil | 8.0±7.5 |
| Whole grains | 9.9±8.2 |
| Juice | 1.65±2.8 |
| Meat | 4.4±3.3 |
| Poultry | 2.0±1.4 |
| Fish | 2.1±1.7 |
| Pastries and sweets | 10.0±9.2 |
| Butter and stick margarine | 5.8±7.8 |
| Fried foods | 2.2±2.2 |
| Cheese | 6.6±4.5 |

**Table 2**. Average weekly consumption of each food group

^*^Values are means ± SDs
